# Anti-Diabetic Effects and Anti-Inflammatory Effects of *Laminaria japonica* and *Hizikia fusiforme* in Skeletal Muscle: In Vitro and In Vivo Model

**DOI:** 10.3390/nu10040491

**Published:** 2018-04-16

**Authors:** Sae-ym Kang, Eunyoung Kim, Inhae Kang, Myoungsook Lee, Yunkyoung Lee

**Affiliations:** 1 Dietetics Team, Cheju Halla General Hospital, Jeju 63127, Korea; ksi3379@naver.com; 2 Department of Food Science and Nutrition, Jeju National University, Jeju 63243, Korea; sky90710@naver.com (E.K.); inhaek@jejunu.ac.kr (I.K.); 3 Department of Food and Nutrition, Research Institute of Obesity Sciences, Sungshin Women’s University, Seoul 01133, Korea; mlee@sungshin.ac.kr

**Keywords:** brown seaweeds, anti-diabetic, *Laminaria japonica*, *Hizikia fusiforme*, C2C12 cell, skeletal muscle

## Abstract

*Laminaria japonica* (*LJ*) and *Hizikia fusiforme* (*HF*) are brown seaweeds known to have various health-promoting effects. In this study, we investigated the anti-diabetic effects and possible mechanism(s) of *LJ* and *HF* by using both in vitro and in vivo models. C2C12 myotubes, mouse-derived skeletal muscle cells, treated with *LF* or *HF* extracts were used for the in vitro model, and muscle tissues from C57BL/6N mice fed a high-fat diet supplemented with 5% *LF* or *HF* for 16 weeks were used for the in vivo model. Although both the *LF* and *HF* extracts significantly inhibited α-glucosidase activity in a dose-dependent manner, the *HF* extract had a superior α-glucosidase inhibition than the *LF* extract. In addition, glucose uptake was significantly increased by *LJ*- and *HF*-treated groups when compared to the control group. Phosphorylation of protein kinase B and AMP-activated protein kinase was induced by *LJ* and *HF* in both the vivo and in vitro skeletal muscle models. Furthermore, *LJ* and *HF* significantly decreased tumor necrosis factor-α whereas both extracts increased interleukin (IL)-6 and IL-10 production in lipopolysaccharide-stimulated C2C12 myotubes. Taken together, these findings imply that the brown seaweeds *LJ* and *HF* could be useful therapeutic agents to attenuate muscle insulin resistance due to diet-induced obesity and its associated inflammation.

## 1. Introduction

Obesity causes enlarged adipocytes along with the infiltration of immune cells to induce chronic low-grade systemic inflammation, which further increases the production of free fatty acids and the number of inflammatory cytokines in circulation [1]. It has been more than two decades since the molecular pathways that link inflammation and insulin resistance have been demonstrated [2,3]. Thus, obesity and the inflammation axis are part of a vicious cycle that develops insulin resistance (IR) in various insulin-sensitive tissues, such as the liver, skeletal muscles, and adipose tissues, leading to type 2 diabetes (T2D) [4]. In general, T2D is a chronic disease caused by deficient insulin secretion that is characterised by abnormally increased blood glucose due to reduced insulin secretion and/or insulin sensitivity [2].

Skeletal muscles are the largest tissue in our body and account for the majority of insulin-mediated glucose uptake in the post-prandial state; therefore, they play a pivotal role in maintaining glucose homeostasis [5,6]. Insulin signaling to promote glucose uptake in skeletal muscle is initiated by activating phosphatidylinositol-3 kinase and Akt (Protein Kinase B) [5]. In addition, 5′ adenosine monophosphate-activated protein kinase (AMPK) is another important signaling molecule that promotes intracellular glucose uptake independently from insulin [7]. It is known that AMPK is activated by exercise and anti-diabetic drugs, such as metformin, as well as various phytochemicals [8].

A number of studies regarding the various health-promoting effects of brown seaweeds have been conducted in vitro and in vivo [9,10,11,12,13,14,15,16,17,18,19]. Brown seaweeds contain plenty of carbohydrates, essential amino acids, vitamins, and minerals. In particular, their carbohydrates, such as laminarian and alginic acid, are a form of dietary fiber that delays gastric emptying to reduce the glucose and triglycerides in circulation. Furthermore, brown seaweeds have been reported to contain various phenolic compounds that have anti-oxidant, anti-inflammatory, and anti-carcinogenic effects [12,14,16].

Previously, we demonstrated that mice fed a high-fat diet (HFD, 60% calories from fat) supplemented with four types of brown seaweeds (*Undaria pinnatifida* (*UP*), *Laminaria Japonica* (*LJ*), *Sargassum Fulvellum* (*SF*), and *Hizikia Fusiforme* (*HF*)) for 16 weeks showed that only *LJ* supplementation improved insulin sensitivity when compared to mice fed an HFD only based on an insulin tolerance test [15]. Although a 5% seaweed supplementation did not prevent a long-term HFD-induced obesity, fasting glucose levels were reduced in mice fed HFD + *LJ*, HFD + *SF*, and HFD + *HF* compared to mice fed an HFD. In addition, adipose tissue-specific inflammation was reduced by all four types of brown seaweed supplementation. Furthermore, bone-marrow-derived macrophage (BMDM) cells from mice fed an HFD plus seaweed groups secreted less pro-inflammatory cytokines by lipopolysaccharides (LPS) stimulus than BMDMs from mice fed an HFD, suggesting less-primed immune cells by seaweed consumption. Therefore, we concluded that dietary consumption of brown seaweeds led to attenuated IR by partially reducing adipose tissue inflammation in a long-term HFD-induced obese mouse model. Since muscle accounts for most in vivo glucose disposal [6], we further examined the most potent brown seaweeds among four previously tested brown seaweeds and *LJ* and *HF* for their anti-diabetic and anti-inflammatory effects and possible mechanism(s) in in vitro and in vivo skeletal muscle models in this study.

## 2. Material and Methods

### 2.1. Sample Preparations

*Laminaria Japonica* (*LJ*) and *Hizikia Fusiforme* (*HF*) were purchased from a traditional market in Jeju, South Korea from December 2014 to March 2015. The powder of the freeze-dried seaweeds was prepared as described in [20]. Brown seaweed extracts were prepared through consecutive maceration of the seaweed powder with Milli-Q water at room temperature for 24 h at a ratio of 1:60 (volume, sample:water). Then, the obtained extracts were filtered using Whatman^®^ filter paper and the filtrate was lyophilized to obtain the powdered extract. The pulverized seaweeds were then dissolved and/or diluted in Dulbecco’s Phosphate-Buffered Saline (DPBS, Gibco BRL, Gaithersburg, MD, USA) and utilized in the in vitro experiments.

### 2.2. Total Polyphenol Contents of LJ and HF Extracts

The total polyphenols of the *LJ* and *HF* extracts were determined using the modified Folin–Denis method [21]. The same volume of seaweed extracts (50 μL) and 1 M Folin–Ciocalteu’s phenol reagent (FMD Millipore Corporation, Darmstadt, Germany) were added to each well of a 96-well plate and left at room temperature for 5 min. One hundred μL of 2% Na_2_CO_3_ solution was added to the reaction and incubated for another 30 min at room temperature protected from light using aluminum foil. The absorbance was read at 720 nm at room temperature by a spectrophotometer (Molecular devices, San Jose, CA, USA) and the results were expressed as gallic acid concentration equivalents.

### 2.3. Alpha-Glucosidase Inhibitory Activities of LJ and HF Extracts

To measure the inhibition of α-glucosidase activity, which is a key enzyme in carbohydrate digestion located on the brush-border surface membranes of intestinal cells, a method reported by Watanabe et al. [22] was performed. In brief, 0.7 UNIT yeast α-glucosidase (Sigma, St. Louis, MO, USA) and 5 mM p-nitrophenyl-α-d-glucopyranoside (P-NPG, Sigma, St. Louis, MO, USA) were dissolved in 100 mM phosphate buffer (pH 7.0) containing 0.2% bovine serum albumin (BSA, Thermo Fisher Scientific, Lombard, IL, USA) and 0.02% NaN_3_ (Sigma, St. Louis, MO, USA), which were used as the enzyme and substrate for the reaction, respectively. In a 96-well plate, 50 μL of the enzyme solution and 10 μL of the *LJ* and *HF* extracts were incubated at 37 °C for 5 min, and the absorbance was measured at 405 nm. Subsequently, 50 μL of P-NPG (5 mM) was added as a substrate and incubated further at room temperature for 5 min, and the change in absorbance at 405 nm was measured. The absorbance was measured using an ELISA Microplate reader, and the α-glucosidase inhibitory activity was calculated using the following equation; [*Alpha*-glucosidase inhibitory activities = (optical density (OD) of sample − OD of control)/OD of control].

### 2.4. Animals and Diets

Male C57BL/6N mice (5 weeks old) were purchased from Orient Bio Co. (Sungnam-si, Korea) and housed individually. Animals were maintained in a temperature- (21 ± 2 °C) and humidity- (50 ± 20%) controlled room with a 12 h dark–light cycle. They were acclimatized for 1 week before the experiment, and the mice were randomly grouped and freely fed with a high-fat diet (60% kcal% fat (D12492), HFD) or a modified HFD supplemented with 5% freeze-dried *LJ* or *HF* as previously described in [15]. All experimental animals were handled according to the guidelines of the Jeju National University Guide for the Care and Use of Laboratory Animals (#2014-0004).

### 2.5. Cell Culture

The mouse-derived C2C12 myoblast cell line was purchased from American Type Culture Collection (ATCC, Manassas, VA, USA) and cultured in high-glucose Dulbecco’s modified eagle medium (DMEM) supplemented with 10% fetal bovine serum (FBS), 1% penicillin/streptomycin (P/S), and 1% l-glutamine at 37 °C in 5% CO_2_. To differentiate C2C12 myoblasts from myotubes, cells were seeded in a 6-well plate or 12-well plate at concentrations of 2.5 × 10^5^ cells/well or 1.0 × 10^5^ cells/well, respectively. When they reached 90–100% confluence, the growth media was changed to a differentiation media, DMEM supplemented with 2% horse serum (HS) and 1% P/S, then the cells were differentiated for an additional 5 to 7 days. All materials for cell culture were purchased from Gibco (BRL, Gaithersburg, MD, USA).

### 2.6. Cell Viability

A 3-(4,5-dimethyl thiazol-2-yl)-2,5-diphenyl tetrazolium bromide (MTT) assay was performed to investigate potential cellular toxicity by the *LJ* and *HF* extracts in C2C12 myotubes. Cells were seeded into 24-well plates (1.0 × 10^5^ cells/well) and differentiated to myotubes as described above. *LJ* and *HF* extracts were treated at various concentrations (25, 50, 100, 200, and 400 μg/mL) in differentiated C2C12 myotubes for 24 h. Next, 100 μL of MTT solution (2 mg/mL) was added to each well of the plate and incubated for another 4 h at 37 °C in humidified air and 5% CO_2_. After removing the media from a plate, 100 μL of DMSO (dimethyl sulfoxide) was added to a well to dissolve the formazan product from cells and the absorbance was measured at 540 nm by using an ELISA microplate reader.

### 2.7. Measurement Glucose (2-NBDG) Uptake

Effects of brown seaweed extracts on glucose uptake in C2C12 muscle cells were measured by using a fluorescent D-glucose analogue, 2-deoxy-2-[(7-nitro-2,1,3-benzoxadizaol-4-yl) amino]-d-glucose (2-NBDG, Life technologies, Rockford, IL, USA), in C2C12 cells as described by Park et al. (2014) and with some modifications [23]. In brief, cells were seeded and differentiated into 12-well plates (1.0 × 10^5^ cells/well). Differentiated C2C12 muscle cells were cultured in serum-free low-glucose DMEM medium for 12 h. One hundred μg/mL of *LJ* or *HF* extracts were treated, followed by treating 10 μM 2-NBDG for 24 h protected from light. Insulin (Sigma, St. Louis, MO, USA) was used as a positive control. At the end of the treatment, cells were washed twice with cold DPBS to remove glucose that did not enter the cells and then treated with 1% Triton-X-100 (Sigma, St. Louis, MO, USA) for 5–10 min. Cells were then transferred to 96 black well fluorescence plates. The fluorescence intensity of cellular 2-NBDG in each well was measured at an excitation wavelength of 485 nm and an emission wavelength of 528 nm using a SpectraMax^®^ i3 plate reader (Molecular Devices, San Jose, CA, USA).

### 2.8. Western Blotting Analysis

C2C12 muscle cells and mouse muscle (quadriceps) tissue from a previous animal study [15] were utilized to determine the activity of two brown seaweeds on intramuscular proteins in vitro and in vivo, respectively.

Differentiated C2C12 muscle cells treated with LJ or HF were lysed by adding 100 L of lysis buffer and centrifugation was carried out for 15 min at 14,000 rpm and 4 °C to obtain the supernatant used for in vitro samples. For in vivo samples, quadriceps of C57BL/6N male mice fed an HFD, HFD + *LJ*, or HFD + *HF* for 16 weeks were collected as described elsewhere [15]. Protein from the frozen tissue samples was isolated as described previously [23]. Briefly, the quadriceps (100 mg) was pulverized in liquid nitrogen and processed in 1 mL of lysis buffer (20 mM Tris-HCL (pH 7.4), 5 mM Na_4_P_2_O_7_, 10 mM NaF, 100 mM NonidetP-40, 1% NaVO_4_, 0.5% EZ block), and centrifuged for 20 min at 14,000 rpm and 4 °C. Supernatants were obtained and used for Western blotting analysis.

Proteins from the in vitro and in vivo samples were quantified using a protein assay kit (Thermo Fisher Scientific, Lombard, IL, USA). Proteins (30 g/25 L) were then separated by SDS–PAGE (SDS–polyacrylamide gel electrophoresis) and transferred to nitrocellulose membranes (Amersham Co., Baden-Württemberg, Germany). Membranes were processed with 5% blocking buffer (Bio-Rad, Hercules, CA, USA) for 2 h followed by the indicated antibody and horseradish peroxidase-coupled anti-species antibodies. Proteins were visualized by enhanced chemiluminescence and quantified by densitometry using Fusion solo (Vilber lourmat, Baden-Württemberg, Germany). All antibodies were purchased from Cell signaling technology (Boston, MA, USA).

### 2.9. Inflammation Cytokines Detection by ELISA

Differentiated C2C12 muscle cells were treated with *LJ* or *HF* extracts. After 3 h of *LJ* or *HF* treatment, the cells were stimulated with lipopolysaccharides (LPS, Sigma, St. Louis, MO, USA) at a concentration of 100 ng/mL for 24 h. Collected media was centrifuged and subjected to inflammatory cytokines measurement. ELISA performed as kits for detecting TNF-α, IL-6 (BD PharMingen, San Jose, CA, USA), and IL-10 (R&D system, Minneapolis, MN, USA) were used for the experiment and proceeded according to the protocol. Absorbance was measured at 450–570 nm by an ELISA microplate reader.

### 2.10. Statistical Analysis

All of the data were expressed as the means ± standard error (SE) and statistical calculations were performed using ANOVA (one-way analysis of variance) followed by Tukey’s multiple comparison test. Results were considered significant if *p* < 0.05 (Graph pad Prism Version 6.0, La Jolla, CA, USA).

## 3. Results

### 3.1. Total Polyphenol Contents and α-Glucosidase Inhibitory Activities of LJ and HF Extracts

**Figure 1:**
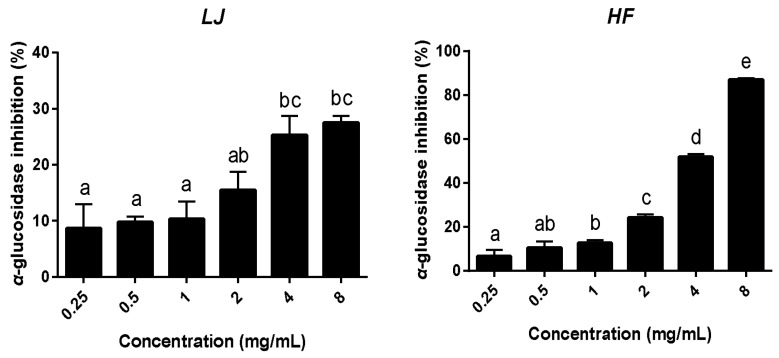
α-glucosidase inhibitory activities of *LJ* and *HF*. Data are represented as the mean ± SEM of three independent experiments. ^a^^–^^e^: Values that do not share the same superscript are significantly different by ANOVA (*p* < 0.05).

The total polyphenol contents of the *LJ* and *HF* extracts were 2.084 and 3.215 μg gallic acid/mL, respectively, where the *HF* extracts contained higher polyphenol contents than the *LF* extracts (Table 1). The alpha-glucosidase inhibitory activities of the two seaweed extracts were measured as described in the methods (Figure 1). As a result, both the *LJ*- and *HF*-treated groups appeared to have inhibited α-glucosidase activity in a dose-dependent manner. In particular, the *HF* extract appeared to have superior α-glucosidase inhibition effects to those of the *LF* extract since the *HF*-treated group had significantly higher α-glucosidase inhibition (87.02%) when compared to the *LJ* extract (27.53%) at an 8 mg/mL concentration.

### 3.2. Effects of LJ and HF Extracts on Cell Viability C2C12 Myotubes

**Figure 2:**
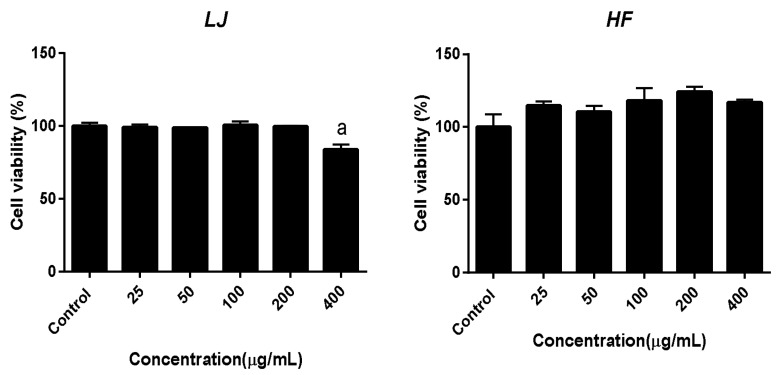
Effects of *LJ* and *HF* extracts on cell viability in C2C12 muscle cells. Data are represented as add the mean ± SEM of three independent experiments. ^a^: Values that do not share the same superscript are significantly different by ANOVA (*p* < 0.05).

Cell viability was measured by the MTT assay. Differentiated C2C12, mouse myotube cells, were treated with 0, 25, 50, 100, 200, and 400 μg/mL of *LJ* or *HF* extracts for 24 h. As shown in Figure 2, there was no significant reduction of cell viability by different doses of *LJ* extract except for the 400 μg/mL concentration of the *LJ*-treated group. On the other hand, none of the *HF*-extract-treated groups appeared to have a significant reduction of cell viability as they showed no toxicity in mouse myotubes. Based on this cellular toxicity assay of two brown seaweed extracts, 100 μg/mL of *LJ* or *HF* was used for the rest of the in vitro experiments.

### 3.3. Effects of LJ and HF Extracts on 2-NBDG Uptake in C2C12 Cells

**Figure 3:**
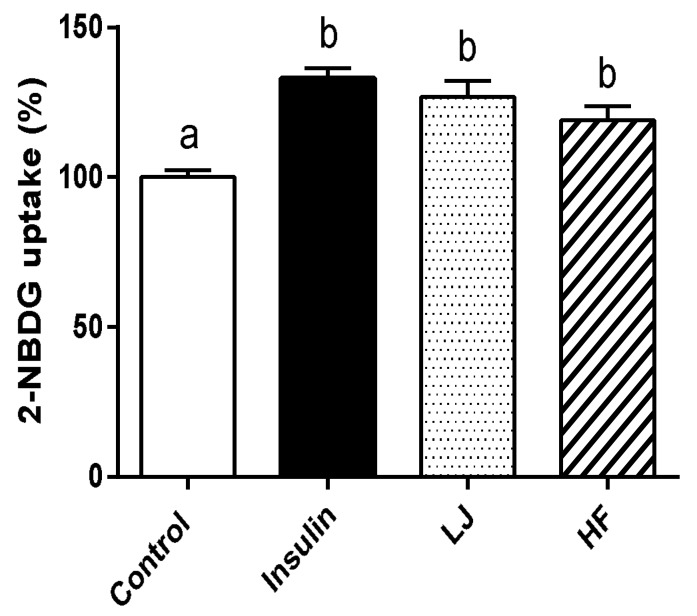
Effects of *LJ* and *HF* extracts on glucose uptake in C2C12 muscle cells. Data are represented as the mean ± SEM of three independent experiments. ^a,b^: Values that do not share the same superscript are significantly different by ANOVA (*p* < 0.05).

To establish whether *LJ* and *HF* stimulated the incorporation of glucose into C2C12 cells, we evaluated its effects on the uptake of a fluorescent d-glucose analogue, 2-NBDG. As shown in Figure 3, the stimulatory effect of *LJ* and *HF* (100 μg/mL) on 2-NBDG uptake was observed in C2C12 muscle cells after 24 h of *LJ* or *HF* treatment. Compared to the control group, the percentage of glucose uptake by *LJ* and *HF* was 126.76% and 119.24%, respectively, which was comparable to the insulin-treated group as a positive control.

### 3.4. Effects of LJ and HF on Insulin-Signaling-Involved Proteins in Skeletal Muscle: In Vitro and In Vivo

**Figure 4:**
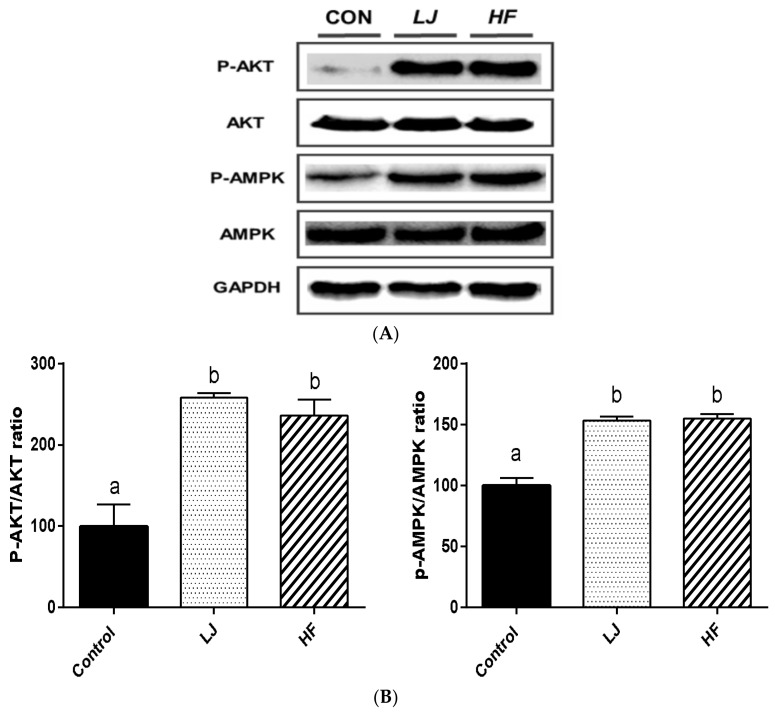
Effects of *LJ* and *HF* extracts on the activation of Akt and AMPK in C2C12 muscle cells. Data are represented as the mean ± SEM of three independent experiments. (**A**) Representative immunoblot analysis of phosphorylation and total Akt and AMPK; (**B**) Quantitative results of (**A**). ^a,b^: Values that do not share the same superscript are significantly different by ANOVA (*p* < 0.05). CON = control.

**Figure 5:**
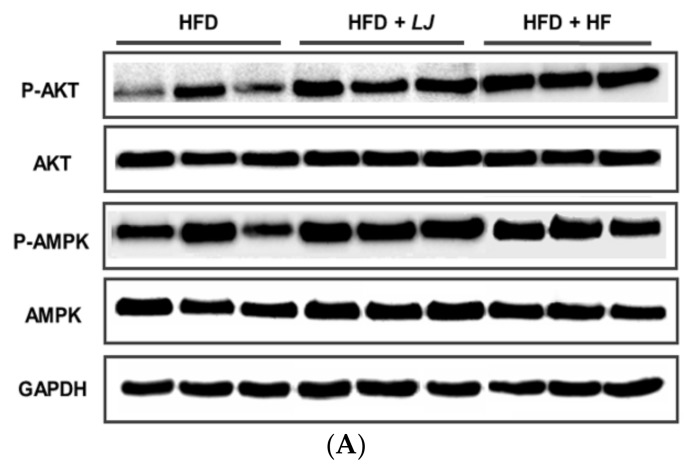

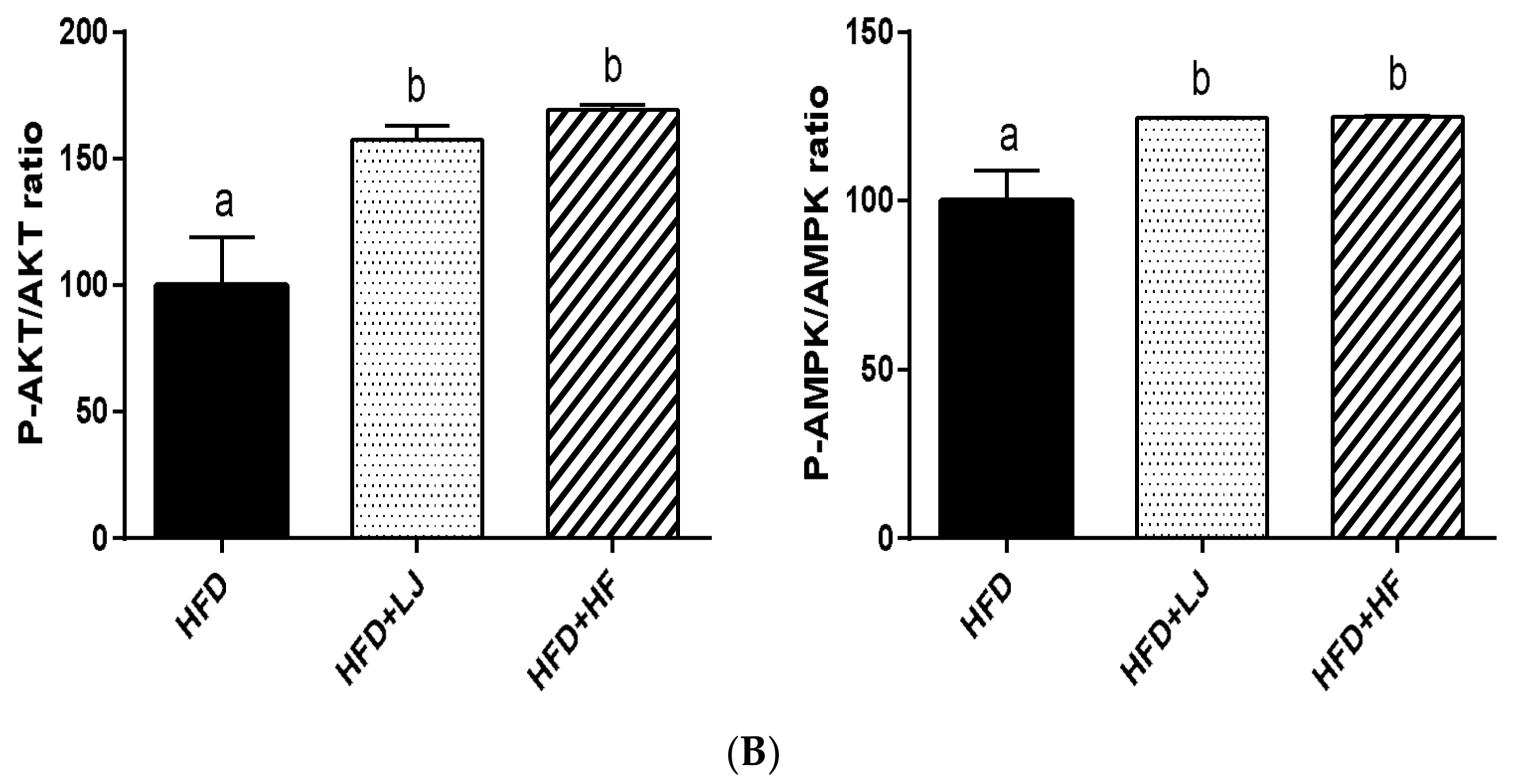
Effects of *LJ* and *HF* on the activation of Akt and AMPK in skeletal muscle from mice fed a high-fat diet (HFD) for 16 weeks. Data are represented as the mean ± SEM (*n* = 5). (**A**) Representative immunoblot analysis of phosphorylation and total Akt and AMPK; (**B**) Quantitative results of (**A**). ^a,b^: Values that do not share the same superscript are significantly different by ANOVA (*p* < 0.05).

Figure 4 and Figure 5 show representative Western blotting analysis results from in vitro and in vivo muscle samples treated with *LJ* or *HF*, respectively. Phosphorylation of Akt (p-Akt) was significantly increased by *LJ* or *HF* treatment in the C2C12 muscle cells when compared to the control group as well as the phosphorylation of AMPK (Figure 4A,B). The muscle tissues of C57BL/6N mice fed an HFD supplemented with 5% *LJ* or *HF* for 16 weeks also had significantly enhanced phosphorylation of Akt and AMPK when compared to those of the mice fed an HFD only (Figure 5A,B). Thus, the activities of insulin signaling pathway related proteins, such as Akt and AMPK, by *LJ* or *HF* were improved in the in vitro and in vivo muscle system in a similar manner.

### 3.5. Effects of LJ and HF Extracts on Inflammatory Response in C2C12 Muscle Cells Stimulated with LPS

To establish the potential anti-inflammatory effects of *LJ* and *HF* extracts in muscle cells, LPS-stimulated C2C12 cells were treated with *LJ* or *HF*, and TNF-α, IL-6, and IL-10 were measured by ELISA (Table 2). With LPS stimuli, all three inflammatory cytokines were significantly increased when compared to the non-LPS-treated C2C12 group, which was not detectable. Dramatically increased TNF-α by LPS were significantly decreased by *LJ* or *HF*, whereas LPS-induced IL-6 and IL-10 were further increased by *LJ* or *HF* in the C2C12 muscle cells.

## 4. Discussion and Conclusions

It has now been well-reported that obesity-induced chronic inflammation causes insulin resistance in muscle, liver, and adipose cells, which then contribute a systemic insulin resistance as well as producing pro-inflammatory cytokines, such as TNF-α and IL-6, for a feed-forward mechanism [1]. Among the insulin-targeted tissues, skeletal muscle plays a critical role in maintaining glucose homeostasis. In fact, the synthesis of muscle glycogen accounts for most of the whole-body glucose uptake, suggesting the pivotal role of skeletal muscle in blood glucose regulation in both normal and diabetic individuals [24]. Anti-inflammatory and anti-diabetic effects of brown seaweeds and their extracts have been reported in previous studies [10,15,18,25,26]. In addition, our previous study demonstrated that dietary consumption of brown seaweeds led to attenuated IR partially by reducing adipose tissue inflammation in a long-term HFD-induced obese animal model [15]. However, this study particularly further evaluated the anti-inflammatory and anti-diabetic effects of two of the most potent brown seaweeds, *LJ* and *HF*, in skeletal muscles by utilizing in vitro and in vivo models. Our results demonstrated that both the *LF* and *HF* extracts effectively inhibited α-glucosidase activity and improved glucose uptake in C2C12 myotubes partially due to the activation of Akt and AMPK, which reconciled well with the results in muscle tissue from mice fed an HFD along with 5% *LJ* or *HF* supplementation.

α-Glucosidase is a key enzyme in carbohydrate digestion and is located on the brush-border surface membranes of intestinal cells [27]. Along with α-amylase action, α-glucosidase degrades disaccharides into simple sugars that cause elevation in blood glucose levels. Therefore, profound inhibition of intestinal α-glucosidase is an effective approach that can be applied in the management of T2D [28]. In this study, we showed that both *LJ* and *HF* extracts reduced *α*-glucosidase activity in a dose-dependent manner. Seaweeds are known to have various bioactive compounds with great potential in the pharmaceuticals, food, and biomedical industries. In particular, brown seaweeds are a rich source of biological compounds, including oligosaccharides and polyphenolic compounds [14,29]. Previous studies have demonstrated that butyl-isobutyl-phthalate in *LJ* and sulfoquinovosyldiacylglycerol in *HF* had inhibitory effects on *α*-glucosidase activity [30,31]. In addition, polyphenols can also inhibit *α*-glucosidase activity [32]. Although further chemical analysis of *LJ* and *HF* is warranted, the inhibitory effects of *α*-glucosidase activity shown in this study accounted for the combinatory effects of various bioactive compounds in brown seaweeds.

Muscle glucose uptake quantitatively accounts for more than 90% of whole-body glucose uptake [6]; therefore, affecting muscle glucose uptake effectively can be an ideal anti-diabetic drug. Both *LJ* and *HF* extracts significantly increased glucose uptake in C2C12 myotube cells in this study. Hao et al. demonstrated that oligomannuronate increased muscle glucose uptake in a dose-dependent manner [29]. Fucoxanthin is a carotenoid of brown seaweed, and Maeda et al. showed that the anti-diabetic effects of fucoxanthin-rich wakame (*Undaria pinnatifida*, *UP*) lipids on a high fat diet induced obesity in mice by enhancing GLUT4 expression in skeletal muscle [10].

Akt, protein kinase B, is one of the serine/threonine protein kinases and is mainly expressed in insulin-sensitive tissues, such as the liver, muscle, and adipose tissues [33]. It is well-known that reduced Akt activation is associated with insulin resistance as well as the dysfunction of glucose transport [34]. Another insulin-signaling-related enzyme, AMPK (5′ adenosine monophosphate-activated protein kinase), acts as an energy sensor that regulates metabolic processes in various tissues to maintain glucose homeostasis [35]. We observed that both of the insulin signaling pathway related proteins Akt and AMPK were significantly activated in skeletal muscle cells and tissues by the *LJ* and *HF* extracts as well as *LJ* and *HF* supplementation in diet-induced obese mice, respectively. A previous study by Kang et al. demonstrated that an extract of the brown seaweed *Ecklonia cava* improved insulin sensitivity of streptozotocin-induced type 1 diabetes mellitus rats by activating AMPK and Akt signaling pathways [17]. They also confirmed that the same phenomenon was true in C2C12 skeletal muscle cells. In fact, the *Ecklonia cava* methanol extract contained various phlorotannin derivates, such as phloroglucinol, eckol, fucodiphloroethol G, phlorofucofuroeckol A, dieckol, 7-phloro eckol, and 6,6′-bieckol [36]. Among these phlorotannin derivates, dieckol in brown seaweeds appeared to be a potent candidate for these beneficial health effects based on the work of other groups, although a detailed analysis needs to be performed [13,18,37].

IL-6 is produced from not only immune cells, but also nonimmune cells and may exert biologically significant effects on a variety of tissues. Especially in muscle physiology, IL-6 plays a dual role in regulating insulin sensitivity [38]. That is, although IL-6 is typically known as a pro-inflammatory cytokine, it can actually improve insulin sensitivity in a tissue-specific manner [39]. For example, Steensberg et al. demonstrated that skeletal muscle dramatically increased IL-6 expression during contraction, and this produced IL-6 was released when muscle glycogen was reduced [40]. Furthermore, exercise decreased TNF-α but increased IL-6, showing that these two pro-inflammatory cytokines work independently in skeletal muscle tissue [41]. On the other hand, when relatively low levels of IL-6 were infused directly in muscle in vivo, it resulted in significant muscle atrophy [42]. Finally, IL-10 is known to be positively correlated with insulin sensitivity. In fact, T2D individuals have reduced IL-10 levels in their circulation [43,44]. Thus, this may explain the observation in this study that *LJ* and *HF* extracts significantly increased IL-6 and IL-10, but decreased TNF-α, expression in LPS-stimulated C2C12 muscle cells, suggesting the use of *LJ* and *HF* as potential therapeutic agents.

The limitations of the study included the following: (1) the physiological condition of high-fat diet fed obese mice with IR could not be completely mimicked in C2C12 muscle cells, including the high levels of free fatty acids and pro-inflammatory cytokines; and (2) components of seaweeds extracts vary depending on what solvents are used. In this study, we utilized water extracts of *LJ* or *HF* in the in vitro model and pulverized freeze-dried seaweeds in the in vivo model. Further studies are warranted to narrow down a potential insulin-signaling cascade influenced by *LJ* and *HF* extracts.

In conclusion, the brown seaweeds *LJ* and *HF* have strong potential to positively affect glucose homeostasis by inhibiting α-glucosidase activity, increasing muscle glucose uptake, and activating insulin-signaling-related proteins. Furthermore, *LJ* and *HF* extracts influence inflammatory cytokines production in skeletal muscle cells.

## Figures and Tables

**Table 1:** Total polyphenol contents of *Laminaria japonica* (*LJ*) and *Hizikia fusiforme* (*HF*) extracts.

Sample	Total Polyphenol Contents (μg Gallic Acid/mL)
*LJ*	2.084 ± 0.01 ^(1)^
*HF*	3.215 ± 0.08

^(1)^ Values are presented as the mean ± standard error of the mean (SEM) of three independent experiments.

**Table 2:** Effects of *LJ* and *HF* extracts on cytokine production in lipopolysaccharide (LPS)-stimulated C2C12 myotube cells.

Sample	TNF-α Level (pg/mL)	IL-6 Level (pg/mL)	IL-10 Level (pg/mL)
LPS	30.68 ± 4.68 ^(1),(2),b^	62.13 ± 2.22 ^a^	0.28 ± 0.57 ^a^
LPS + *LJ*	15.03 ± 4.46 ^a^	433.15 ± 2.64 ^c^	5.61 ± 1.07 ^c^
LPS + *HF*	14.82 ± 3.09 ^a^	106.00 ± 6.75 ^b^	3.77 ± 1.50 ^b^

^(1)^ Value are presented as the mean ± SEM of three independent experiments. ^(2)^ ^a^^–^^c^: Values that do not share the same superscript are significantly different by ANOVA (*p* < 0.05).

## Abbreviations

2-NBDG	2-deoxy-2-[(7-nitro-2,1,3-benzoxadizaol-4-yl) amino]-d-glucose
Akt	Protein Kinase B
AMPK	5′ adenosine monophosphate-activated protein kinase
BMDM	bone marrow derived macrophage
DMEM	Dulbecco’s modified eagle medium
GAPDH	glyceraldehyde-3-phosphate dehydrogenase
*HF*	*Hizikia fusiforme*
HFD	high fat diet
IL	interleukin
IR	insulin resistance
*LJ*	*Laminaria japonica*
LFD	low fat diet
LPS	lipopolysaccarides
MTT	3-(4,5-dimethyl thiazol-2-yl)-2,5-diphenyl tetrazolium bromide
*SF*	*Sargassum Fulvellum*
T2D	type 2 diabetes
TNF-α	tumor necrosis factor-α
*UP*	*Undaria pinnatifida*
